# GWASBrewer: An R Package for Simulating Realistic GWAS Summary Statistics

**DOI:** 10.1002/gepi.22594

**Published:** 2024-10-06

**Authors:** Jean Morrison

**Affiliations:** ^1^ Department of Biostatistics University of Michigan Ann Arbor Michigan USA

**Keywords:** evaluation, GWAS summary statistics, mendelian randomization, polygenic risk score, simulation

## Abstract

Many statistical genetics analysis methods make use of GWAS summary statistics. Best statistical practice requires evaluating these methods in realistic simulation experiments. However, simulating summary statistics by first simulating individual genotype and phenotype data is extremely computationally demanding. This high cost may force researchers to conduct overly simplistic simulations that fail to accurately measure method performance. Alternatively, summary statistics can be simulated directly from their theoretical distribution. Although this is a common need among statistical genetics researchers, no software packages exist for comprehensive GWAS summary statistic simulation. We present GWASBrewer, an open source R package for direct simulation of GWAS summary statistics. We show that statistics simulated by GWASBrewer have the same distribution as statistics generated from individual level data, and can be produced at a fraction of the computational expense. Additionally, GWASBrewer can simulate standard error estimates, something that is typically not done when sampling summary statistics directly. GWASBrewer is highly flexible, allowing the user to simulate data for multiple traits connected by causal effects and with complex distributions of effect sizes. We demonstrate example uses of GWASBrewer for evaluating Mendelian randomization, polygenic risk score, and heritability estimation methods.

## 1 Introduction

In recent years there has been a proliferation of statistical methods that use effect estimates and standard errors (summary statistics) from genome‐wide association studies (GWAS) to infer interesting biological parameters. These include methods for estimating heritability (Bulik‐Sullivan, Loh, et al. 2015; Gazal et al. 2017; Speed, Holmes, and Balding 2020; Zhang et al. 2021), genetic correlation (Bulik‐Sullivan, Finucane, et al. 2015; Ning, Pawitan, and Shen 2020; Wu et al. 2022), causal effects via Mendelian randomization (MR) (Zheng et al. 2017; Sanderson et al. 2022; Slob and Burgess 2020), and polygenic risk scores (Maier et al. 2018; Lloyd‐Jones et al. 2019; Zabad, Gravel, and Li 2022; Ge et al. 2019). A common challenge in the development of all methods applied to genetic data is conducting simulation studies that adequately mimic properties of real data. Overly simplistic simulations may mis‐represent the expected behavior of a method. One of the most realistic strategies to generate summary statistics is the full‐data simulation procedure. In this procedure, genotype data are generated by sampling haplotypes from a reference panel, from a population‐genetic model, or from a correlated binomial distribution (Ritchie and Bush 2010; Montana 2005; Peng et al. 2015). Phenotype data are generated according to particular (usually additive) genetic model and finally association estimates are computed for each variant. The resulting summary statistics can then be passed to the method or methods being evaluated.

A major drawback of the full‐data simulation method is that it is computationally intensive and involves production and storage of large quantities of data. Some effort can be saved by using the same set of genotype data in every simulation replicate and only regenerating phenotype data. However, the step of computing association estimates requires performing up to millions of linear regressions. This problem is compounded when data from multiple GWAS are required. This high computational demand may motivate investigators to limit the scope of their simulations or make simplifying assumptions that could alter the conclusions of the experiment. For example, many MR methods have been evaluated by generating data for a small number of pre‐selected independent variants (Slob and Burgess 2020; Zhao et al. 2019; Verbanck et al. 2018). However, when these methods are applied in practice, variants must be selected from genome‐wide data. This step can add bias to the MR estimate that is not captured by overly simplistic simulations. Another common simplification is to assume that there is no LD between variants. However, this results in much sparser genetic signal than would be expected in real data and can lead to over‐optimism about the accuracy of some methods.

When evaluating methods that only require GWAS summary statistics, it is possible to directly simulate summary statistics without generating individual level data by sampling estimates from their asymptotic multivariate normal distribution. This strategy has been used by Morrison et al. (2020) among others. The direct summary statistic simulation method can preserve many of the important features of real GWAS data, such as LD, with a fraction of the computational resources required by the full‐data simulation method. However, despite the large number of methods developed each year for analysis of GWAS summary statistics and the common need to perform simulation and benchmarking studies, there are no well‐documented R packages implementing the direct summary statistic simulation method for a range of scenarios. To fill this gap, we developed the R package GWASBrewer. The goal of GWASBrewer is to generate data that are as realistic as possible from a flexible model that can accommodate many simulation needs. Features of GWASBrewer include the ability to simulate data for multiple traits with specified causal relationships, for variants in linkage disequilibrium, allowing for arbitrary amounts of overlap between GWAS samples, and allowing for flexible specification of variant effect size distributions and heritability models.

In Section 2, we describe how GWASBrewer samples GWAS summary statistics. Section 3, describes the interface and specific features of GWASBrewer. In Section 4 we demonstrate that GWASBrewer accurately mimics the distribution of summary statistics obtained through full‐data simulation. Finally, in Section 6, we demonstrate the use of GWASBrewer to test MR methods, heritability estimation methods, and polygenic risk score estimation methods.

## 2 Simulating GWAS Summary Statistics

### 2.1 Summary Statistics for a Single Trait

We first describe simulation of summary statistics for a single continuous trait, Y. Let $G_{1},\ldots ,G_{J}$ be genetic variants with correlation R (frequently referred to as the linkage disequilibrium (LD) matrix). We assume that all genetic variants are bi‐allelic and in Hardy‐Weinberg equilibrium. Together, these assumptions mean that $G_{j}$ is a binomial random variable with frequency $f_{j}$, and variance $\mathrm{Var}\left(G_{j}\right)\equiv v_{j}=2f_{j}\left(1-f_{j}\right)$.

In a GWAS, Y and $G=\left(G_{1},\ldots ,G_{J}\right)$ are measured for N individuals yielding observations $\left(y_{1},g_{1}\right),\ldots ,\left(y_{N},g_{N}\right)$. The association between $G_{j}$ and Y is then estimated using linear regression if Y is continuous, or logistic regression if Y is binary. Most modern GWAS include age, sex, and principal components of the genoytpe matrix as covariates, and may also use a mixed model to account for relatedness between samples. We make a simplifying assumption that GWAS samples are unrelated, sampled from a homogeneous randomly mating population, and that effect estimates are estimated via simple linear regression. That is,

(1) $${\hat{\beta }}_{j}=\frac{\sum_{i=1}^{N}\left(g_{i,j}-{\bar{g}}_{j}\right)\left(y_{i}-\bar{y}\right)}{\sum_{i=1}^{N}{\left(g_{i,j}-{\bar{g}}_{j}\right)}^{2}},$$

(2) $${\hat{s}}_{j}^{2}\equiv {\hat{se}}^{2}\left({\hat{\beta }}_{j}\right)=\frac{\frac{1}{N-2}\sum_{i=1}^{N}{\left(y_{i}-\bar{y}\right)}^{2}}{\sum_{i=1}^{N}{\left(g_{i,j}-{\bar{g}}_{j}\right)}^{2}}-\frac{1}{N-2}{\hat{\beta }}_{j}^{2},$$

where $\bar{y}$ and ${\bar{g}}_{j}$ are sample means of Y and $G_{j}$ respectively. Although our summary statistic simulation procedure is motivated by simple linear regression, it can also be used to simulate data from covariate adjusted GWAS, assuming that no heritable traits are adjusted for and there is no residual confounding. This topic is discussed in greater detail in Supporting Information S1: Section 1.

Zhu and Stephens (2017) demonstrate that the joint distribution of $\hat{\beta }={\left({\hat{\beta }}_{1},\ldots ,{\hat{\beta }}_{J}\right)}^{⊤}$ is well approximated by a multivariate normal distribution,

(3) $$\hat{\beta }\sim N\left({\mathrm{SRS}}^{-1}\beta^{\left(\mathrm{joint}\right)},\mathrm{SRS}\right),$$

where S is a J×J diagonal matrix with jth diagonal entry equal to $s_{j}=\sqrt{\frac{\mathrm{Var}\left(Y\right)}{v_{j}N}}$ and $\beta^{\left(\mathrm{joint}\right)}$ is the vector of population joint associations between variants and Y. For simplicity and without loss of generality, we assume that Var(Y)=1, so $s_{j}$ depends only on $f_{j}$ and N. The joint association between $G_{j}$ and Y is the linear association conditional on all other variants. If we assume a purely additive genetic model,

(4) $$Y=\sum_{j=1}^{J}\beta_{j}^{\left(\mathrm{joint}\right)}G_{j}+\epsilon .$$

In this case, $\beta^{\left(\mathrm{joint}\right)}$ is the vector of causal effects. In GWASBrewer, joint effects are simulated from a scale‐family distribution. By default, this is a point‐normal distribution, however, it is possible to specify other alternatives. GWASBrewer chooses the scale parameter so that Y has a user‐specified expected heritability. Optionally, effects can be scaled so that heritability is exact using the h2_exact option. More detail on the distribution of effect sizes is given in Section 2.3.

GWASBrewer simulates GWAS estimates, $\hat{\beta }$, by directly sampling from the model in Equation (3) given $\beta^{\left(\mathrm{joint}\right)},S$, and R. This process is very fast when R is block diagonal with moderately sized blocks. Standard error estimates, ${\hat{s}}_{j}$, can optionally be simulated as well (see Supporting Information S1: Section 1). The LD matrix, R, and allele frequencies $f_{j}$ can be specified by the user, or GWASBrewer will default to generating data with no LD. The package includes one LD matrix and set of allele frequencies as built‐in data objects. These were estimated from chromosome 19 in the European subset of 1000 Genomes data and broken into blocks using LDetect regions (Berisa and Pickrell 2016). GWASBrewer does not require that the provided LD matrix contain the number of variants to be simulated. Instead, it repeats the provided LD pattern as many (possibly fractional) times as necessary to produce the desired number of variants. This means that genome‐sized data can be generated from a smaller LD pattern and that the size of the simulated genome can be changed without generating a new reference LD object. However, requiring the LD pattern to be broken into blocks does prevent modeling of long‐range LD.

### 2.2 Summary Statistics for Multiple Traits

**Figure 1:**
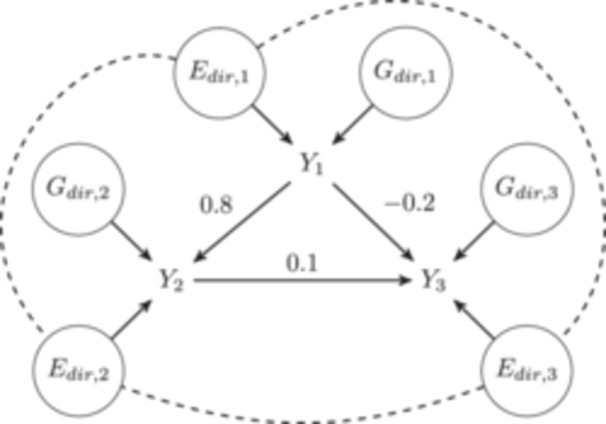
DAG specified by the direct effects matrix in Equation (5). Nodes in circles represent direct genetic and environmental effects. Direct environmental components of traits may be correlated (dashed lines) while direct genetic components are always independent.

Many methods including MR, GenomicSEM, and cross‐trait genetic correlation estimation, involve estimation of relationships between multiple traits. GWASBrewer can facilitate evaluation of these methods by simulating summary statistics for a set of K traits, $Y_{1},\ldots ,Y_{K}$ with specified genetic and environmental relationships. GWASBrewer can simulate summary statistics for an arbitrary number of continuous traits that are related to each other by a specified linear structural equation model (SEM) corresponding to a directed, acyclyc graph (DAG). The SEM for K traits is specified by the user as $D^{\left(\mathrm{dir}\right)}$, a K×K matrix of direct effects in which $D_{k,k^{\prime }}^{\left(\mathrm{dir}\right)}$ gives the direct effect of $Y_{k}$ on $Y_{k^{\prime }}$. For example, the DAG in Figure 1 corresponds to the direct effect matrix,

(5) $$D^{\left(\mathrm{dir}\right)}=\left(\begin{array}{lll} 0 & 0.8 & -0.2\\ 0 & 0 & 0.1\\ 0 & 0 & 0 \end{array}\right),$$

and the linear SEM,

$$\left(\begin{array}{l} Y_{1}\\ Y_{2}\\ Y_{3} \end{array}\right)=D^{\left(\mathrm{dir}\right)⊤}\left(\begin{array}{l} Y_{1}\\ Y_{2}\\ Y_{3} \end{array}\right)+\left(\begin{array}{l} \epsilon_{1}\\ \epsilon_{2}\\ \epsilon_{3} \end{array}\right).$$

In this SEM, $\epsilon_{k},k=1,2,3$ include both direct genetic effects and direct environmental effects. In other words $\epsilon_{k}=G_{\mathrm{dir},k}+E_{\mathrm{dir},k}$ as illustrated in Figure 1. We assume that direct genetic effects are mutually independent and independent of environmental effects. However, we do not require that direct environmental effects are mutually independent of each other.

To simulate summary statistics for all K traits, GWASBrewer performs four steps. (1) Calculate the total variance of the direct genetic components. (2) Simulate direct effects for each variant‐trait pair by sampling from up to K different scale‐family distributions. (3) Calculate the total joint effect of each variant on each trait. (4) Simulate effect estimates and estimated standard errors using a multivariate extension of (3). Here we briefly describe each of these steps.

For the first and third steps, we must first compute the matrix of total trait effects. For example, in the DAG in Figure 1, the total effect of $Y_{1}$ on $Y_{3}$ is 0.8⋅0.1−0.2=−0.12. In a linear SEM corresponding to a DAG, total effects can be calculated from the matrix of direct effects as

(6) $$D^{\left(\mathrm{tot}\right)}=D^{\left(\mathrm{dir}\right)}{\left(I-D^{\left(\mathrm{dir}\right)}\right)}^{-1},$$

where I is the K×K identity matrix (Bollen 1987). At this stage, we can also verify that the user‐supplied $D^{\left(\mathrm{dir}\right)}$ corresponds to an acyclic graph. If this is not the case, either $\left(I-D^{\left(\mathrm{dir}\right)}\right)$ will not be invertable or the resulting $D^{\left(\mathrm{tot}\right)}$ will not have 0's on the diagonal.

Let $h^{2}$ be a user‐supplied vector of trait heritabilities. The total variance of each trait can be decomposed as $\mathrm{Var}(Y_{k})=V_{G\mathrm{dir},k}+V_{G\mathrm{ind},k}+V_{E,k}$, where $V_{G\mathrm{dir},k}$ is the variance due to direct genetic effects, $V_{G\mathrm{ind},k}$ is the variance due to genetic effects mediated by other traits (indirect effects), and $V_{E,k}$ is the variance due to both direct and indirect environmental effects. We assume that $\mathrm{Var}\left(Y_{k}\right)=1$ for all traits, so $h_{k}^{2}=V_{G\mathrm{dir},k}+V_{G\mathrm{ind},k}$. We can calculate that $V_{G\mathrm{ind},k}=\sum_{l=1}^{K}D_{l,k}^{\left(\mathrm{tot}\right)2}V_{G\mathrm{dir},l}$. Therefore, in Step 1, we obtain the direct heritability of each trait as

(7) $$\begin{array}{ccc} & & h^{2}=V_{G\mathrm{dir}}+V_{G\mathrm{ind}}={\left(I+D^{\left(\mathrm{dir}\right)\circ 2}\right)}^{⊤}V_{G\mathrm{dir}}\\ & & V_{G\mathrm{dir}}={\left({\left(I+D^{\left(\mathrm{dir}\right)\circ 2}\right)}^{⊤}\right)}^{-1}h^{2}, \end{array}$$

where superscript ∘2 indicates the element‐wise square.

In Step 2, we sample direct effects so that the total expected variance of direct genetic effects is equal to $V_{G\mathrm{dir}}$. Details of simulation of direct effects are given in Section 2.3. In Step 3, we use the total effects matrix to obtain the total genetic effect of each variant on each trait. Let $\gamma_{j}$ be the K‐vector giving the direct effects of variant j on $Y_{1},\ldots Y_{K}$. The K‐vector of total joint effects of variant $j,\beta_{j}$ is

(8) $$\beta_{j}^{\left(\mathrm{joint}\right)}=\left(I+D^{\left(\mathrm{tot}\right)⊤}\right)\gamma_{j}.$$

In Step 4, we simulate summary statistics using a multivariate extension of Equation (3). The expression in Equation (3) holds for each trait individually. However, it does not provide the covariance between effect estimates for different traits, which depends on the overlap between GWAS samples and the LD between variants. Let $N_{k}$ and $N_{k^{\prime }}$ be GWAS sample size of traits k and $k^{\prime }$, and let $N_{k,k^{\prime }}$ be the number of overlapping samples. The correlation between ${\hat{\beta }}_{k,j}$ and ${\hat{\beta }}_{k^{\prime },j}$ is approximately equal to $\frac{N_{k,k^{\prime }}}{\sqrt{N_{k}N_{k^{\prime }}}}\rho_{k,k^{\prime }}$, where $\rho_{k,k^{\prime }}$ is the population correlation between $Y_{k}$ and $Y_{k^{\prime }}$ (Conneely and Boehnke 2007; Bulik‐Sullivan, Finucane, et al. 2015). More generally, the covariance between effect estimates for any pair of variants and traits is

(9) $$\mathrm{Cov}({\hat{\beta }}_{j,k},{\hat{\beta }}_{j^{\prime },k^{\prime }})\approx \frac{R_{j,j^{\prime }}}{s_{j,k}s_{j^{\prime },k^{\prime }}}\frac{N_{k,k^{\prime }}}{\sqrt{N_{k}N_{k^{\prime }}}}\rho_{k,k^{\prime }},$$

where $R_{j,j^{\prime }}$ is the correlation between variant j and variant $j^{\prime }$ (Conneely and Boehnke 2007). This expression allows us to sample the full J×K matrix of effect estimates. Estimates of the standard errors, ${\hat{s}}_{j,k}$ can be sampled using a multivariate generalization of the strategy used for a single trait (Supporting Information S1: Section 1). By default, the population correlation $\rho_{k,k^{\prime }}$ is calculated assuming that all direct environmental components are independent. However, the user can specify correlation between environmental components either by specifying the overall trait correlation or by specifying the overall correlation of trait environmental components.

### 2.3 Distribution of Direct Variant‐Trait Effects

GWASBrewer supports a wide variety of genetic architectures. By default, direct effects are sparse with a proportion, $\pi_{k}$, being nonzero for each trait. If $\gamma_{j,k}$ is selected to be nonzero, then the standardized effect size, ${\tilde{\gamma }}_{j,k}=\gamma_{j,k}\mathrm{Var}\left(G_{j}\right)$, is drawn from a $N\left(0,\frac{V_{G\mathrm{dir},k}}{\pi_{k}J}\right)$ distribution, giving an expected total direct genetic variance of approximately $V_{G\mathrm{dir},k}$. This approximation assumes that most causal variants are approximately independent, which is reasonable for genome‐wide simulations. Using this strategy, the realized heritability will not exactly equal the input expected heritability, however, if the h2_exact option is used, effects will be scaled after sampling to give exactly the desired heritability.

GWASBrewer has options that allow the user to control both which variants are causal, and the distribution of effect sizes, including permitting exact specification of causal variants and effect sizes. To control which variants are causal, the user can specify a J×K matrix with elements giving the probability that each variant has a direct effect on each trait. If these probabilities are set to 0 or 1, the user can deterministically select effect and non‐effect variants. Alternatively, variant‐specific probabilities can be used to allow the probability of being an effect variant to depend on variant‐level features or to create regions of the genome with different densities of effect variants.

To specify the effect distribution, the user can specify any function for sampling from a scale family distribution (technical details in Section 3). We provide built‐in utilities for generating effect size distribution functions to sample from a mixture of normal distributions and for using a set of effects that is fixed up to a scalar. GWASBrewer allows for each trait to have a different effect size distribution function. It also allows the effect size distribution function to depend on variant‐level annotations which can be passed to the simulation function separately.

## 3 Implementation and Interface

### 3.1 The sim_mv Function

The primary user‐facing function in GWASBrewer is sim_mv. The arguments of sim_mv can be divided into three groups. The first describes the traits, the second describes the GWAS study design, and the third describes the distribution of direct effects of variants on traits. Trait‐related arguments include the matrix of direct effects, G, heritability, h2, and optionally either the observational or environmental correlation of traits, R_obs or R_E. Study design‐related arguments include sample size, N, number of variants to simulate, J, an optional LD pattern R_LD and an optional vector of allele frequencies af. The sample size argument can accept multiple formats and allows specification of sample overlap. Finally, effect distribution arguments include the proportion of causal variants, pi, an optional user‐supplied effect size function or list of functions, snp_effect_function, and an optional dataframe of annotations that may be used by the user‐specified effect function, snp_info. sim_mv produces an object of class sim_mv containing summary statistics as well as true marginal and joint effects, true heritability, environmental covariance, and trait correlation. A complete description of inputs and outputs for sim_mv is given in Supporting Information S1: Section 2.

### 3.2 Auxiliary Functions

The GWASBrewer package contains several functions to assist with common tasks. These include utilities for LD‐pruning (sim_ld_prune), extracting LD proxies (sim_ld_proxy), and extracting the LD matrix for a set of variants (sim_extract_ld). GWASBrewer also includes two functions that make it possible to resample summary satistics or individual level data with the same joint effects as an initial object produced by sim_mv, resample_sumstats, and resample_inddata. The resample_sumstats function can be used to simulate new GWAS of the same trait, possibly with different sample sizes or different LD patterns. The resample_inddata function generates individual level genotype and phenotype data from the effects in a previously generated simulation object. In the examples below, we show how this function can be used to test the predictive ability of a polygenic risk score. The resample_inddata function uses utilities from the HapSim R package (Montana 2005) to simulate genotypes with the specified LD structure. It then simulates phenotypes from Equation (4) assuming environmental effects are normally distributed with covariance given by Sigma_E in the original simulation object. Optionally, resample_inddata can efficiently calculate the linear regression summary statistics for the generated data.

## 4 Accuracy

We used simulations to verify that summary statistics generated by GWASBrewer follow the same distribution as summary statistics generated from individual level data. We first generated effect sizes for 10 variants in LD and two traits. The first trait has two causal variants, the second trait has three causal variants, with no common causal variants and no causal effect between traits. For both traits, causal effects from variants included explain 0.4% of trait variance. We then used resample_sumstats and resample_inddata to generate 1000 realizations of directly sampled summary statistics and 1000 realizations of summary statistics computed from individual‐level data. In both cases, we specified an observational correlation between traits of 0.7. The sample size for Trait 1 was 8000 and the sample size for Trait 2 was 10,000, with 2000 individuals in both samples. Code for performing these simulations is provided in a supplemental file.

**Figure 2:**
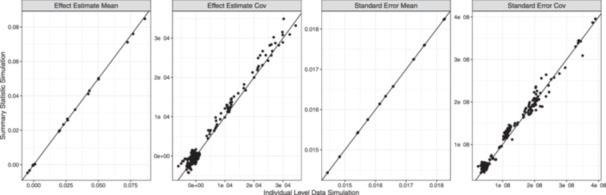
Comparison of summary statistics from the full data simulation method and the direct summary statistic simulation method used by GWASBrewer. Panels compare the mean and covariance of simulated effect standard error estimates. For mean plots, each point corresponds to one variant‐trait pair. For covariance plots, each point corresponds to one pair of variant‐trait pairs.

Figure 2 compares the mean, standard deviation, and correlation of effect estimates either directly sampled or derived from individual‐level data. We find that all values are very similar between the two methods. We additionally constructed quantile–quantile (Q–Q) plots comparing the distribution of effect estimates and standard error estimates between the two methods for all variants and both traits as well as Q–Q plots comparing products of effect estimates and standard error estimates across traits. All Q–Q plots were consistent with the hypothesis that the two methods generate data with the same distribution (Supporting Information S1: Section 3).

## 5 Computational Cost

Simulating summary statistics directly provides benefits in terms of both compute time and data storage. Table 1 displays median CPU time required for summary statistic and individual level simulation using two different LD structures. In the first, AR1‐500, the LD structure consists of a single 500 variant AR1 matrix with correlation parameter 0.9. This block is replicated by GWASBrewer to cover the desired number of variants. The second LD structure, EUR, uses the built‐in European LD structure. Using a smaller LD repeat lowers the computational cost for GWASBrewer because SVD computations can be shared across repeats. We conducted simulations for a single trait with 10,000 variants and sample size ranging from 500 to 5000 as well as simulations with sample size 100,000 and number of variants ranging from 500 to 5000. As expected, computation time for summary statistic simulation does not depend on the number of samples, while time for individual‐level data simulation increases approximately linearly with sample size. Generation of genotypes using HapSim is, by far, the slowest step in these simulations, accounting for 90%–99% of individual‐level simulation time. Time for individual‐level data simulation could be greatly reduced by sampling from existing haplotypes, or using another more efficient simulation strategy.

**Table 1:** CPU time and object size for summary statistic and individual‐level data simulation.

			Summary statistics				Individual‐level data				
N	J	LD type	Total time	$\hat{\beta }$	$\hat{s}$	Size (MB)	Total time	Genotypes	Phenotypes	LR	Size (MB)
500	10 k	AR1‐500	0.64	0.41	0.25	2.00	5.1	4.9	0.06	0.10	20
1 k	10 k	AR1‐500	0.62	0.38	0.24	2.00	8.9	8.6	0.11	0.20	40
5 k	10 k	AR1‐500	0.63	0.38	0.23	2.0	22	21	0.24	1.60	200
100 k	5000	AR1‐500	0.41	0.27	0.16	0.08	20	19	0.26	1.2	210
100 k	1 k	AR1‐500	0.40	0.27	0.15	0.21	40	37	0.47	2.6	410
100 k	5 k	AR1‐500	0.47	0.31	0.15	1.0	190	170	2.4	19	2000
100 k	1 M	AR1‐500	11	8.2	3.0	210	$38,000^{†}$	$33,000^{†}$	$470^{†}$	$3900^{†}$	$40,000^{†}$
500	10 k	EUR	9.4	4.4	4.9	1.4	49	48	0.14	0.1	20
1 k	10 k	EUR	9.4	4.40	4.9	1.4	53	52	0.17	0.2	40
5 k	10 k	EUR	9.3	4.3	4.9	1.4	67	65	0.35	1.7	200
100 k	500	EUR	0.81	0.43	0.37	0.08	24	22	0.25	1.50	210
100 k	1 k	EUR	1.1	0.51	0.56	0.15	45	42	0.46	2.7	410
100 k	5 k	EUR	4.4	2.1	2.2	0.73	220	200	2.4	19	2000
100 k	1 M	EUR	44	25	18	210	$44,000^{†}$	$39,000^{†}$	$470^{†}$	$3900^{†}$	$40,000^{†}$

*Note: N* indicates GWAS sample size. *J* indicates the number of variants. LD Type describes the LD structure. Time is reported in seconds and object size in megabytes. Columns $\hat{\beta }$and $\hat{s}$list the component times for simulation of effect estimates and standard error estimates respectively. Columns genotypes, phenotypes, and LR give the component times for simulation of the genotype matrix, phenotypes, and computation of simple linear regression summary statistics. All values listed are medians over 10 replicates. Values marked with † are extrapolated from observed values at the same sample size assuming a linear relationship with number of variants.

The benefit of using a summary statistic only simulation strategy is most apparent for large sample sizes and large numbers of variants. On our system, simulating summary statistics for 100 k individuals and 1 million variants required only 11 s using the AR1‐500 LD pattern or 44 seconds using the EUR LD pattern, and the total size of the generated data were only 210 MB. We estimated the time and data storage requirements for individual‐level data simulation at these larger parameters from results in smaller data sets with the same sample size. Using our implementation, the linear regression step alone would occupy more than an hour of CPU time per data set, and each data set would occupy 40 GB when stored as a standard R matrix. To reduce the amount of data held in RAM, it would be necessary to write and read data to files, requiring bespoke code, and, likely, access to a compute cluster. By contrast, direct summary statistic simulation using GWASBrewer can be performed easily on a personal computer using package functions.

Complete system information for the computer used to perform these experiments and simulation details are provided in Supporting Information S1: Section 4 and Supplementary Code (Supporting Information S2). More extensive compute time results are provided in Table S1.

## 6 Examples

### 6.1 Simulating Multiple Traits From a DAG

**Figure 3:**
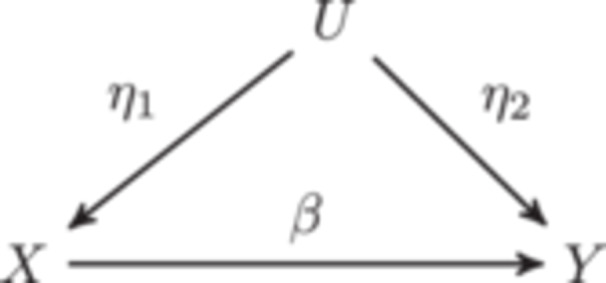
DAG for Mendelian randomization example.

In this example, we simulate data that could be used to evaluate an MR method in the presence of heritable confounding. We simulate GWAS data for an exposure, X, and outcome, Y, variable with a common heritable cause, U, shown in Figure 3. We then use package functions to identify exposure instruments that can be used for MR estimation. In this case, our DAG includes three traits, but we only need summary statistics for the exposure and the outcome. We can avoid simulating unneeded summary statistics for U by setting the sample size for U to zero.

We first set the direct effects matrix which is passed to the G parameter of sim_mv,

$$D^{\left(\mathrm{dir}\right)}=\left(\begin{array}{lll} 0 & \beta & 0\\ 0 & 0 & 0\\ \eta_{1} & \eta_{2} & 0 \end{array}\right).$$

In this case, the traits are listed in the order of X,Y,U.


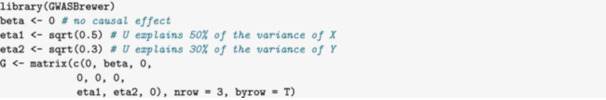


To make the simulations as realistic as possible, we generate data with LD and simulate the standard errors. We use the matrix format for sample size to indicate that there are 40,000 individuals in the exposure GWAS, 60,000 in the outcome GWAS, with 10,000 individuals overlapping. The last row and column of the sample size matrix are zero because we don't need summary statistics for U.


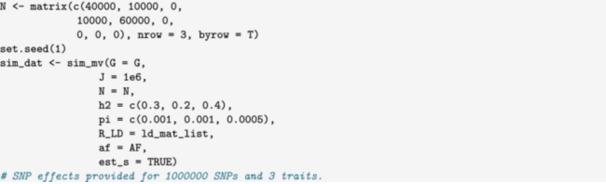


The summary statistics that we need are stored in beta_hat and s_estimate. No summary statistics are produced for U, because the sample size for U was given as 0.

In a typical MR analysis, we select the top independent variants associated with the exposure to use as instruments. We can do this using the sim_ld_prune function. Below, we use this function to LD prune using the p‐value for X, the first trait ( *p*‐value = 1). The function returns a vector of variant indices.


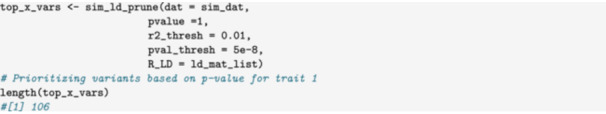


Alternatively, we could simulate a three sample MR study, in which there are two separate GWAS for X, one for selection and one for testing. To generate data for a third selection sample, we can use the resample_sumstats function. For the selection GWAS, we only need data for X so we set sample sizes for the other two traits to zero. In this case, we will use the same LD pattern for the selection GWAS as for the testing GWAS, but we could have chosen to do otherwise. Alternatively, we could generate all three GWAS in a single call to sim_mv by creating a fourth trait $X^{\prime }$ and setting the effect of X on $X^{\prime }$ to 1. This method would not allow the selection GWAS to have a different LD pattern from the other studies, but would allow for a partially overlapping cohort.


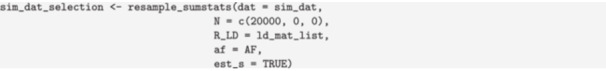


### 6.2 Advanced Effect Size Options

In this example, we demonstrate some advanced options for specifying the distribution of variant effects. These are applicable to scenarios which require more control over the location of causal variants or the distribution of causal variant effect size than provided by the default options. For example, several alternative heritability models have been proposed including the LDAK (Speed, Holmes, and Balding 2020) and LDSC (Bulik‐Sullivan, Loh, et al. 2015) models. Under the LDSC model, expected SNP heritability is constant across variants, while in the LDAK model, $E\left[h_{j}^{2}\right]\propto w_{j}{\left(f_{j}\left(1-f_{j}\right)\right)}^{0.75}$, where $h_{j}^{2}$ is the heritability contributed by variant j, and $w_{j}$ is a weight that depends on the LD score of the variant. Proposals have also been made for estimating heritability enrichment by variant annotations (Zhang et al. 2021; Gazal et al. 2017). To evaluate these methods in simulations, we would need to simulate data with different effect distributions, potentially dependent on variant annotations.

#### 6.2.1 Controlling the Location of Causal Variants

The location of causal variants is controlled through the pi parameter which can be given as a scalar, a vector with length equal to the number of traits, or a matrix. If pi is a scalar or vector, each variant has equal probability of being causal for a given trait. The matrix specification allows the user to specify individual probabilities of being causal for each variant‐trait pair. In the example code below, matrix specification is used to restrict causal variants to the first half of the genome. Recall that the parameter *G* = 1 can be used to simulate one trait.


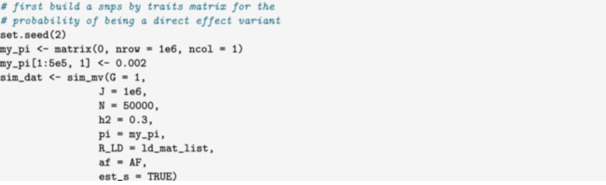


In a more complex example, we let the probability that a variant is causal be proportional to its LD score:


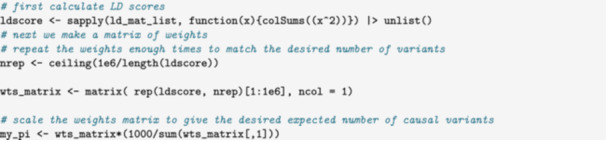


The weight matrix my_pi can then be passed to the pi argument of sim_mv.

#### 6.2.2 Controlling the Distribution of Variant Effects

The distribution of direct effects can be controlled with the snp_effect_function argument. This argument can accept a single function that will be used for all traits or a list of K functions. Functions passed to snp_effect_function should accept three parameters: *n* , sd, and snp_info and return a vector of *n* standardized effect sizes $\left(a_{1},\ldots ,a_{n}\right)$ such that $\sum_{i=1}^{n}E\left[a_{i}^{2}\right]=\mathrm{sd}$. Many simple distribution functions will ignore the snp_info argument, which can be used to allow effect sizes to depend on an annotation or the allele frequency.

GWASBrewer contains two helper functions for forming variant effect functions for common distribution families, the scale mixture of normals (mixnorm_to_scale_fam), and effects specified directly up to a constant (fixed_to_scale_fam). In the scale mixture of normals, the direct effect of variant j on trait k is drawn from a distribution

$$\gamma_{j,k}\sim \sum_{m=1}^{M}\tau_{m}N\left(0,\sigma_{m}^{2}\right).$$

The set of mixture parameters $\tau_{1},\ldots ,\tau_{M}$ and variance parameters $\sigma_{1}^{2},\ldots ,\sigma_{M}^{2}$ specify the distribution. To generate causal effects from a two‐part mixture in which $\sigma_{2}^{2}=10\sigma_{1}^{2}$ with 90% of causal variants drawn from the first component and 10% from the second, we use


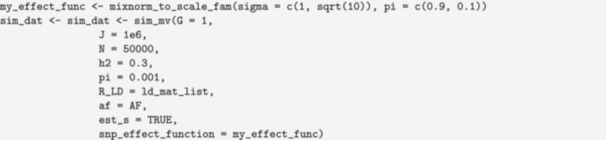


Note that there are generally multiple ways to get the same result. In example above, 0.1% of variants are causal. We could achieve equivalent results by including a 0 component in the scale mixture of normals and setting pi to 1.

Alternatively, the user can write their own variant effect functions. Here we show an example in which effect size depends on two annotations, mimicking a simplified version of the LDAK annotation model. We generate data in which

$$E\left[h_{j}^{2}\right]\propto {\left(f_{j}\left(1-f_{j}\right)\right)}^{0.75}\left(\alpha_{0}+\alpha_{1}A_{1,j}+\alpha_{2}A_{2,j}\right).$$

For our example, annotations $A_{1}$ and $A_{2}$ will be generated randomly. In a real application, these annotations could be real genomic annotations such as chromatin accessibility or distance to promoter. Annotations are supplied to sim_mv through the snp_info argument. These are passed to the variant effect function with an added column, AF containing allele frequency. We first write a variant effect function to give the desired distribution using $\alpha_{0}=0.9,\alpha_{1}=0.1$, and $\alpha_{2}=0.13$.


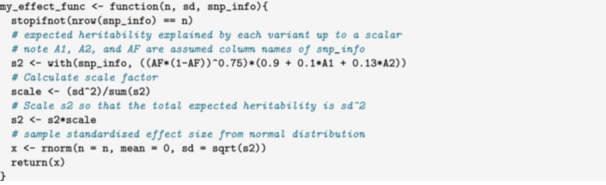


Next we generate random annotations. If we are using LD, the number of rows in the annotation data frame should match the total size of the LD pattern.


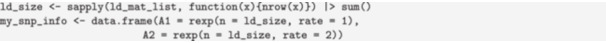


Finally, we can run sim_mv passing my_effect_func to snp_effect_function and my_snp_info to snp_info.

### 6.3 Simulating Individual Level Data

In the last example, we simulate data that could be used to evaluate accuracy of a polygenic risk score (PRS) constructed from GWAS results. This simulation demonstrates use of the resample_inddata to generate individual level genotype and phenotype data.

First, we simulate summary statistics for the discovery GWAS that will be used to build the PRS.


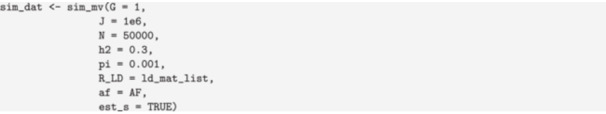


After constructing the PRS using any method, we need to simulate testing data. We will use the same LD structure used to generate discovery data. Using a different LD structure would allow us to measure cross‐ancestry PRS transportability under the assumption that only LD and not effect sizes differ between populations. To generate indivdual level data, we use the resample_inddata function. The code below generates 1000 test samples. By default, resample_inddata uses hapsim (Montana 2005) to simulate genotypes. However, alternative genotype simulation functions can be supplied to the sim_func argument.


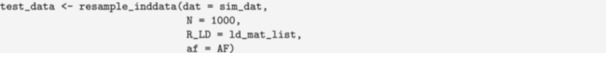


In a simulation study, we would need to repeat each of these steps many times. This will be more efficient if we generate only one set of test genotypes to be used in every iteration. To facilitate this, resample_inddata has three alternative modes, one in which it generates genotype data only, and one in which it accepts genotype data and simulates only phenotypes, and the mode shown above which generates both genotype and phenotype data.

To generate only genotype data, we simply omit the dat argument and add the J argument to specify the desired number of variants to generate.


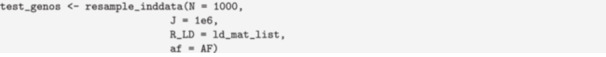


To generate phenotype data only, we can pass in the previously generated genotypes.


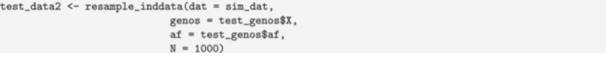


## 7 Discussion

We have introduced GWASBrewer, a software package for efficiently simulating GWAS summary data for multiple traits, allowing for LD between variants, correlation between traits, and any amount of sample overlap. We demonstrated that summary statistics produced by GWASBrewer have the same distribution as summary statistics produced through individual level data simulation. Simulating summary statistics requires substantially less CPU time and less data storage than simulating individual level data. As a result, GWASBrewer can facilitate more in‐depth and realistic simulations, leading to more accurate characterizations of method performance. GWASBrewer has potential utility for evaluating a wide range of statistical methods, including MR, colocalization, and heritability estimation methods. GWASBrewer allows for flexible specification of effect size distributions, and provides useful utility functions to aid in downstream evaluation of data. GWASBrewer also provides a unique feature of simulating standard error estimates, rather than returning only the true standard error of simulated effect sizes.

Although we have attempted to design GWASBrewer to be flexible enough to address many simulation needs, it has several limitations, some of which we hope to address in future releases. GWASBrewer currently only supports continuous traits related through linear structural equation models, and does not model gene‐environment or gene–gene interactions. Additionally, GWASBrewer does not include confounding bias from factors such as uncorrected population structure, and generates data only for unrelated samples. In future releases, we plan to expand support for binary traits, with linear relationships specified on the liability scale and expand the error model to allow for residual confounding.

## Conflicts of Interest

The author declares no conflicts of interest.

## Supporting information

Supplementary Information

Supplementary Information

Supplementary Information

## Data Availability

All code replicating simulations presented in this paper is available in Supplementary Code. The GWASBrewer software package is publicly available on Github at https://jean997.github.io/GWASBrewer/. Data sharing is not applicable to this article as no new data were created or analyzed in this study.

## References

[gepi22594-bib-0001] Berisa, T. , and J. K. Pickrell . 2016. “Approximately Independent Linkage Disequilibrium Blocks in Human Populations.” Bioinformatics (Oxford, England) 32, no. 2: 283–285. 26395773 10.1093/bioinformatics/btv546 PMC4731402

[gepi22594-bib-0002] Bollen, K. A. 1987. “Total, Direct, and Indirect Effects in Structural Equation Models.” Sociological Methodology 17: 37–69.

[gepi22594-bib-0003] Bulik‐Sullivan, B. , H. K. Finucane , V. Anttila , et al. 2015. “An Atlas of Genetic Correlations Across Human Diseases and Traits.” Nature Genetics 47, no. 11: 1236–1241. 26414676 10.1038/ng.3406 PMC4797329

[gepi22594-bib-0004] Bulik‐Sullivan, B. , P.‐R. Loh , and H. K. Finucane , et al. 2015. “LD Score Regression Distinguishes Confounding From Polygenicity in Genome‐Wide Association Studies.” Nature Genetics 47: 291–295. 25642630 10.1038/ng.3211 PMC4495769

[gepi22594-bib-0005] Conneely, K. N. , and M. Boehnke . 2007. “So Many Correlated Tests, so Little Time! Rapid Adjustment of P Values for Multiple Correlated Tests.” The American Journal of Human Genetics 81, no. 6: 1158–1168. 17966093 10.1086/522036 PMC2276357

[gepi22594-bib-0006] Gazal, S. , H. K. Finucane , N. A. Furlotte , et al. 2017. “Linkage Disequilibrium‐Dependent Architecture of Human Complex Traits Shows Action of Negative Selection.” Nature Genetics 49, no. 10: 1421–1427. 28892061 10.1038/ng.3954 PMC6133304

[gepi22594-bib-0007] Ge, T. , C. Y. Chen , Y. Ni , Y. C. A. Feng , and J. W. Smoller . 2019. “Polygenic Prediction Via Bayesian Regression and Continuous Shrinkage Priors.” Nature Communications 10, no. 1: 1–10. 10.1038/s41467-019-09718-5 PMC6467998 30992449

[gepi22594-bib-0008] Lloyd‐Jones, L. R. , J. Zeng , J. Sidorenko , et al. 2019. “Improved Polygenic Prediction By Bayesian Multiple Regression on Summary Statistics.” Nature Communications 10, no. 5086: 1–11. 10.1038/s41467-019-12653-0 PMC6841727 31704910

[gepi22594-bib-0009] Maier, R. M. , Z. Zhu , S. H. Lee , et al. 2018. “Improving Genetic Prediction by Leveraging Genetic Correlations Among Human Diseases and Traits.” Nature Communications 9, no. 1: 1–17. 10.1038/s41467-017-02769-6 PMC5841449 29515099

[gepi22594-bib-0010] Montana, G. 2005. “Hapsim: A Simulation Tool for Generating Haplotype Data With Pre‐Specified Allele Frequencies and LD Coefficients.” Bioinformatics 21, no. 23: 4309–4311. 16188927 10.1093/bioinformatics/bti689

[gepi22594-bib-0011] Morrison, J. , N. Knoblauch , J. H. Marcus , M. Stephens , and X. He . 2020. “Mendelian Randomization Accounting for Correlated and Uncorrelated Pleiotropic Effects Using Genome‐Wide Summary Statistics.” Nature Genetics 52, no. 7: 740–747. 32451458 10.1038/s41588-020-0631-4 PMC7343608

[gepi22594-bib-0012] Ning, Z. , Y. Pawitan , and X. Shen . 2020. “High‐Definition Likelihood Inference of Genetic Correlations Across Human Complex Traits.” Nature Genetics 52, no. 8: 859–864. 32601477 10.1038/s41588-020-0653-y

[gepi22594-bib-0013] Peng, B. , H.‐S. Chen , L. E. Mechanic , et al. 2015. “Genetic Data Simulators and Their Applications: An Overview.” Genetic Epidemiology 39, no. 1: 2–10. 25504286 10.1002/gepi.21876 PMC4804465

[gepi22594-bib-0014] Ritchie, M. D. , and W. S. Bush . 2010. “Genome Simulation Approaches for Synthesizing in Silico Datasets for Human Genomics.” Advances in Genetics 72: 1–24. 21029846 10.1016/B978-0-12-380862-2.00001-1

[gepi22594-bib-0015] Sanderson, E. , M. M. Glymour , M. V. Holmes , et al. 2022. “Mendelian Randomization.” Nature Reviews Methods Primers 2, no. 1: 1–21. 10.1038/s43586-021-00092-5 PMC7614635 37325194

[gepi22594-bib-0016] Slob, E. A. W. , and S. Burgess . 2020. “A Comparison of Robust Mendelian Randomization Methods Using Summary Data.” Genetic Epidemiology 44, no. 4: 313–329. 32249995 10.1002/gepi.22295 PMC7317850

[gepi22594-bib-0017] Speed, D. , J. Holmes , and D. J. Balding . 2020. “Evaluating and Improving Heritability Models Using Summary Statistics.” Nature Genetics 52, no. 4: 458–462. 32203469 10.1038/s41588-020-0600-y

[gepi22594-bib-0018] Verbanck, M. , C.‐Y. Chen , B. Neale , and R. Do . 2018. “Detection of Widespread Horizontal Pleiotropy in Causal Relationships Inferred From Mendelian Randomization between Complex Traits and Diseases.” Nature Genetics 50: 693–698. 29686387 10.1038/s41588-018-0099-7 PMC6083837

[gepi22594-bib-0019] Wu, Y. , K. S. Burch , A. Ganna , P. Pajukanta , B. Pasaniuc , and S. Sankararaman . 2022. “Fast Estimation of Genetic Correlation for Biobank‐Scale Data.” The American Journal of Human Genetics 109, no. 1: 24–32. 34861179 10.1016/j.ajhg.2021.11.015 PMC8764132

[gepi22594-bib-0020] Zabad, S. , S. Gravel , and Y. Li . 2022 Fast and Accurate Bayesian Polygenic Risk Modeling With Variational Inference.” American Journal of Human Genetics 110: 741–761. 10.1016/j.ajhg.2023.03.009 PMC10183379 37030289

[gepi22594-bib-0021] Zhang, Q. , F. Privé , B. Vilhjálmsson , and D. Speed . 2021. “Improved Genetic Prediction of Complex Traits From Individual‐Level Data or Summary Statistics.” Nature Communications 12, no. 1: 4192. 10.1038/s41467-021-24485-y PMC8263809 34234142

[gepi22594-bib-0022] Zhao, Q. , J. Wang , G. Hemani , J. Bowden , and D. S. Small . 2019. “Statistical Inference in Two‐Sample Summary‐Data Mendelian Randomization Using Robust Adjusted Profile Score.” arXiv:1801.09652 [math, stat].

[gepi22594-bib-0023] Zheng, J. , D. Baird , M.‐C. Borges , et al. 2017. “Recent Developments in Mendelian Randomization Studies.” Current Epidemiology Reports 4: 330–345. 29226067 10.1007/s40471-017-0128-6 PMC5711966

[gepi22594-bib-0024] Zhu, X. , and M. Stephens . 2017. “Bayesian Large‐Scale Multiple Regression With Summary Statistics From Genome‐Wide Association Studies.” Annals of Applied Statistics 11, no. 3: 1561–1592. 29399241 10.1214/17-aoas1046 PMC5796536

