# Supplementary material for: GWASBrewer: An R Package for Simulating Realistic GWAS Summary Statistics
Source: Genet Epidemiol. 2024 Oct 6;49(1):e22594. doi: 10.1002/gepi.22594 (PMC11656154; doi:10.1002/gepi.22594)
Supplement: Supplementary file 1 — Supplementary Information [file GEPI-49-0-s002.pdf]

GWASBrewer: An R Package for Simulating Realistic GWAS Summary Statistics

Jean Morrison
University of Michigan, Department of Biostatistics
jvmorr@umich.edu

1 Summary Statistic Simulation

1.1 Simulation of Standard Errors

In this section we describe the strategy used by `GWASBrewer` to simulate standard error estimates. We begin with two useful results.

Lemma 1.1. *Let* $\mathbf{X}_1, \dots, \mathbf{X}_N$ *be an IID sample from a* d*-variate distribution with mean* $\boldsymbol{\mu}$ *and at least four finite moments. Let* $s_j^2 = \frac{1}{N}\sum_{i=1}^{N}(X_{j,i} - \bar{X}_j)^2$ *be the sample variance in the* j*th dimension, let* $\mathbf{s}^2 = (s_1^2, \dots, s_d^2)^\top$*, and let* $\boldsymbol{\sigma}^2 = (\sigma_1^2, \dots, \sigma_d^2)$ *be the* d*-vector of component-wise variances. Then*

$$\sqrt{n}(\mathbf{s}^2 - \boldsymbol{\sigma}^2) \to N_d(0, \Theta)$$

where Θ *is a* $d \times d$ *covariance matrix with elements*

$$\Theta_{j,j} = 4\mu_j^2\sigma_j^2 - 4\mu_j Cov(X_j, X_j^2) + Var(X_j^2) \quad (1)$$

$$\Theta_{j,j'} = 4\mu_j\mu_{j'}Cov(X_j, X_{j'}) - 2\mu_j Cov(X_j, X_{j'}^2) - 2\mu_{j'}Cov(X_j^2, X_{j'}) + Cov(X_j^2, X_{j'}^2), \quad j \neq j' \quad (2)$$

Proof. The proof of this lemma follows from the multivariate central limit theorem and the multivariate delta method. Without loss of generality, we derive the expressions above for $d = 2$. Let $\mathbf{z} = (\frac{1}{N}\sum_{i=1}^{N} X_{1,i}, \frac{1}{N}\sum_{i=1}^{N} X_{1,i}^2, \frac{1}{N}\sum_{i=1}^{N} X_{2,i}, \frac{1}{N}\sum_{i=1}^{N} X_{2,i}^2)^\top$ and let $g(\mathbf{z}) = (z_2 - z_1^2, z_4 - z_3^2)^\top$ so that $g(\mathbf{z}) = (s_1^2, s_2^2)$. By the multivariate central limit theorem,

$$\sqrt{n}(\mathbf{z} - E[\mathbf{z}]) \to N_4(0, C)$$

$$C = \begin{pmatrix} \sigma_1^2 & Cov(X_1, X_1^2) & Cov(X_1, X_2) & Cov(X_1, X_2^2) \\ Cov(X_1, X_2) & Var(X_1^2) & Cov(X_1^2, X_2) & Cov(X_1^2, X_2^2) \\ Cov(X_1, X_2) & Cov(X_1^2, X_2) & \sigma_2^2 & Cov(X_2, X_2^2) \\ Cov(X_1, X_2^2) & Cov(X_1^2, X_2^2) & Cov(X_2, X_2^2) & Var(X_2^2) \end{pmatrix}$$

The gradient of the function g evaluated at $E[\mathbf{z}]$ is

$$\nabla g(E[\mathbf{z}]) = \begin{pmatrix} -2\mu_1 & 0 \\ 1 & 0 \\ 0 & -2\mu_2 \\ 0 & 1 \end{pmatrix} \quad (3)$$

Thus, by the multivariate delta method,

$$\sqrt{n}(\mathbf{s}^2 - \boldsymbol{\sigma}^2) \to N_2\left(0, \nabla g(E[\mathbf{z}])^\top C \nabla g(E[\mathbf{z}])\right)$$

Some algebra shows that $\nabla g(E[\mathbf{z}])^\top C \nabla g(E[\mathbf{z}])$ is equal to Θ as specified above. □

Corollary 1.1.1. *Let $X_1, \ldots, X_N$ be an IID sample from a one-dimensional distribution with at least four finite moments. Denote the mean, variance, and fourth central moment as μ, σ^2, μ_4 and let $s^2 = \frac{1}{N}\sum_{i=1}^{N}(X_i - \bar{X})$. Then*

$$\sqrt{n}(s^2 - \sigma^2) \to N(0, \mu_4 - \sigma^4).$$

Proof. This is simply the one-dimensional version of the preceding lemma. □

We start by describing simulation of standard error estimates for one trait. Let $\hat{\mathbf{s}}$ be the J-vector of standard error estimates for all variants. Using the expression in Main Text Equation (2), $\hat{s}_j^2$ can be written as

$$\hat{s}_j^2 = \frac{s_Y^2}{(N-2)s_{G_j}^2} - \frac{1}{N-2}\hat{\beta}_j^2$$

where s_Y^2 and $s_{G_j}^2$ are the sample variances of the phenotype Y and variant G_j respectively. We make the simplifying assumption that $\hat{\beta}_j^2$ is independent of s_Y^2 and $s_{G_j}^2$ and approximate the distribution of $\hat{s}_j$ as

$$\hat{s}_j^2 \sim \frac{W_Y}{(N-2)W_{G,j}} - \frac{1}{N-2}\hat{\beta}_j^2$$

where W_Y and $W_{G,j}$ are respectively samples from the asymptotic distributions of s_Y^2 and $s_{G_j}^2$.

To sample from the asymptotic distribution of s_Y^2, we apply the corollary above, assuming that the fourth moment of the phenotype distribution is equal to the fourth moment of a $N(0,1)$ distribution, or 3. Thus, we sample a single realization of $W_Y \sim N(0,2)$. This single draw is shared for all variants because estimates for all variants are made using the same set of individuals.

For the denominator, we need to sample from the distribution of, $\mathbf{s}_G^2$, the vector of sample variances for all J variants. Here we apply the full multivariate version of Lemma 1.1. We assume that the J-vector of genotypes for individual i is the sum of two independent vectors of Bernoulli random variables (a paternal and maternal haplotype), $\mathbf{G}_i = \mathbf{W}_{i,1} + \mathbf{W}_{i,2}$, where $\mathbf{W}_{i,k}$ ($k = 1, 2$) are IID J-dimensional vectors of correlated Bernoulli random variables such that $W_{i,k,j} \sim Bernoulli(f_j)$ and $Cor(W_{i,k,j}, W_{i,k,j'}) = \rho_{j,j'}$ as specified by the LD matrix, for $k \in \{1, 2\}$. From this distribution, we can calculate that

$$\begin{aligned}
&Var(G_j) = 2f_j(1-f_j) \equiv \sigma_{G_j}^2\\
&Cov(G_j, G_j^2) = \sigma_{G_j}^2(1+2f_j)\\
&Var(G_j^2) = \sigma_{G_j}^2\left((1+2f_j)^2 + \sigma_{G_j}^2\right)\\
&Cov(G_j, G_j') = \rho_{j,j'}\sigma_{G_j}\sigma_{j'}\\
&Cov(G_j, G_{j'}^2) = \rho_{j,j'}\sigma_{G_j}\sigma_{G_{j'}}(1+2f_{j'})\\
&Cov(G_j^2, G_{j'}^2) = \rho_{j,j'}\sigma_{G_j}\sigma_{G_{j'}}(1+2f_j)(1+2f_{j'}) + \rho_{j,j'}^2\sigma_{G_j}^2\sigma_{G_{j'}}^2
\end{aligned}$$

Plugging these in to the expressions in (1) and (2) and condensing terms, we obtain

$$\begin{aligned}
NVar(s_{G_j}^2) =& \sigma_{G_j}^2(1-\sigma_{G_j}^2)\\
NCov(s_{G_j}^2, s_{G_j'}^2) =& \rho_{j,j'}\sigma_{G_j}\sigma_{G_{j'}}(1-2f_j)(1-2f_{j'}) + \rho_{j,j'}^2\sigma_{G_j}^2\sigma_{G_{j'}}^2.
\end{aligned}$$

This allows us to sample $\mathbf{W}_G$ from the normal distribution defined by these expressions given allele frequencies and LD matrix. This simulation scheme does not restrict sampled values of $\hat{s}_j^2$ to be positive. In practice, it is extremely unlikely to sample negative values unless N is low and the variant effect size is large. In the event that this does occur, we resample until we obtain a positive value.

The procedure for sampling standard errors for K traits is the same except that we must sample one numerator for each trait, $\mathbf{W}_Y = (W_{Y_1}, \ldots, W_{Y_K}) \sim N_K(\sigma_Y^2, \Theta_Y)$. If there is sample overlap, then Θ_Y is not

diagonal and we again apply the multivariate version of Lemma 1.1. Since all phenotypes have mean zero, we have

$$\Theta_{Y,j,j} = Var(Y_j^2)$$
$$\Theta_{Y,j,j'} = Cov(Y_j^2, Y_{j'}^2), \quad j \neq j'.$$

As previously, we base values for $Var(Y_j^2)$ and $Cov(Y_j^2, Y_{j'}^2)$ on a standardized, normally distributed phenotype. This gives $Var(Y_j^2) = 3$ and $Cov(Y_j^2, Y_{j'}^2) = 2\frac{N_{C,j,j'}}{\sqrt{N_j N_{j'}}}\rho_{Y_j,Y_j'}$ where $\rho_{Y_j,Y_j'}$ is the observational correlation of Y_j and $Y_{j'}$ and $N_{C,j,j'}$ is equal to the number of samples overlapping between the j and j' GWAS study.

1.2 Covariate Adjustment

`GWASBrewer` does not explicitly model covariates. However, `GWASBrewer` outputs can be interpreted as simulations of adjusted linear regression coefficients, assuming that heritable traits were adjusted for. This is because the coefficient on G_j from a multiple linear regression including covariates is equivalent to the coefficient from a simple linear regression after regressing the covariates out of both the genotype and the phenotype. For simulation purposes, the important differences between adjusted and unadjusted linear regression are in the interpretation of parameters. The specified heritability is the heritability of the adjusted phenotype and allele frequencies should be interpreted as *effective* allele frequencies after covariate adjustment. This is because the variance of the adjusted genotype is lower than the variance of the unadjusted genotype. The effective allele frequency is the value $\tilde{f}_j$ such that $2\tilde{f}_j(1-\tilde{f}_j)$ is equal to the variance of the adjusted genotype. We require the assumption that no covariates are heritable traits, because adjusting for heritable traits can induce colliding, which is not modeled by `GWASBrewer`.

The covariate adjusted genotype will not exactly follow a Binomial(2) distribution. However, assumptions about the genotype distribution are only used in simulation of standard errors, where we make use of the third and fourth moments of the genotype distribution. However, we find that the sampling distribution of standard errors is fairly insensitive to violations of these assumptions.

To test the sensitivity of the sampling distribution of standard errors to assumptions about the genotype distribution, we simulated individual-level data for a single variant with a very strongly associated covariate from the data generating model:

$$W \sim N(0,1)$$
$$p_W = \text{expit}\left(-2 + W\right))$$
$$G_j \sim Binom(2, p_W)$$
$$Y \sim N(0.1W + 0.04G, 1).$$

This data generation scheme produces a covariate W such that G and W have correlation about 0.43, which is much larger than would generally be expected in a GWAS. In this model, the distribution of adjusted genotypes is quite far from a binomial distribution, as shown in Figure 1a. In each simulation, we generated data for 10,000 individuals and fit a multiple linear regression with Y as outcome and G and W as predictors. We also computed the residual genotype variance and effective allele frequency. We then performed simulations using `GWASBrewer`, specifying the allele frequency to be equal to the average of observed effective allele frequencies (about 0.14). Figure 1 compares the distributions of effect estimates and standard error estimates from individual level data and those obtained from `GWASBrewer`, indicating no observable differences in distribution over 1000 simulations. Code replicating these simluations can be found in Supplementary Code.

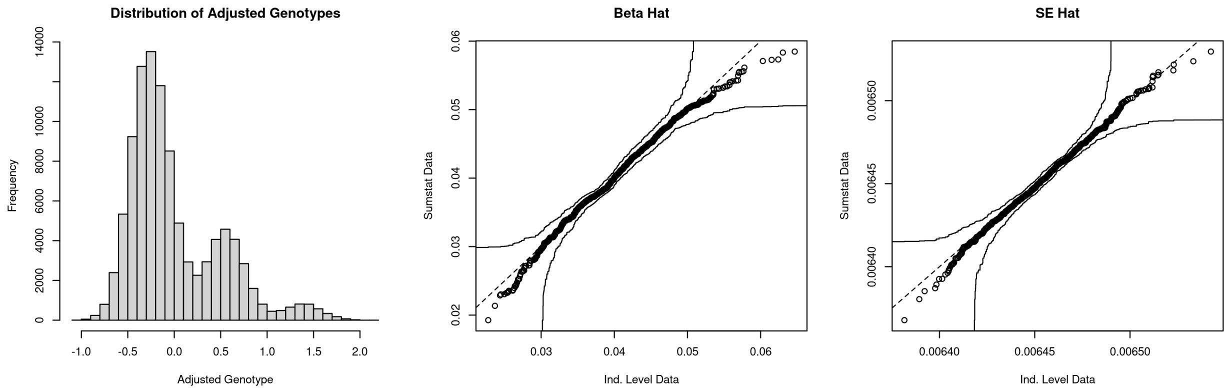

Figure 1: Comparison of summary statistics from covariate adjusted linear regression to summary statistics generated with `GWASBrewer`. Left: Histogram of adjusted genotypes (genotype residuals after regressing out the covariate) for one simulation. Middle: Quantile-quantile plot comparing the distributions of effect estimates from individual level data using multiple linear regression and effect estimates from `GWASBrewer`. Right: Quantile-quantile plot comparing the distributions of standard error estimates from individual level data with multiple linear regression and standard error estimates from `GWASBrewer`. Middle and Right plots depict the results of 1000 simulations.

2 Inputs and Outputs of `sim_mv`

2.1 Input Parameters

<u>Arguments describing the traits</u>

`G` : $K \times K$ matrix of direct trait effects ($D^{(dir)}$).

`h2` : Expected trait heritability. This can be a scalar in which case all traits have the same heritability or a vector of length K.

`R_obs` : Optional $K \times K$ matrix giving the observational correlation between traits. If missing, the correlation is calculated assuming independent direct environmental contributions.

`R_E` : Optional $K \times K$ matrix giving the correlation between environmental components of traits. Only one of `R_obs` and `R_E` should be specified. Note that this is not the same as the correlation between the direct environmental components of traits.

<u>Arguments describing GWAS study design</u>

`N` : GWAS sample size. There are four accepted formats for `N`, scalar, vector, matrix, and dataframe. If `N` is a scalar, all GWAS have the same sample size and there is no sample overlap. If `N` is a vector, there is no sample overlap and each element gives the sample size for the corresponding trait. The matrix and dataframe formats can be used to specify sample overlap. If `N` is a matrix then `N[i,j]` specifies the number of samples in both study `i` and study `j` with `N[i,i]` giving the total sample size of study `i`. Finally, `N` can have dataframe format. In dataframe format, `N` should have columns named `trait_1` ... `trait_[K]` and `N`. The `trait_[k]` columns will be interpreted as logicals and `N` gives the number of samples in each combination of studies (see examples below for more details). For `sim_mv`, matrix and dataframe format contain equivalent information. However, the dataframe format is useful with other package functions.

`J` : Total number of variants to simulate.

R_LD : Optional list of LD blocks. R_LD should be a list with each element describing a block in the block-diagonal LD matrix. Each element can be a matrix, a sparse matrix, or an eigen-decomposition. All elements should be correlation matrices, meaning that they have 1 on the diagonal and are positive definite.

af : Optional vector of allele frequencies. af can be a scalar, vector or function. If af is a function it should take a single argument, n, and return a vector of n allele frequencies. If R_LD is supplied, af must be a vector with length equal to the size of the supplied LD pattern. Otherwise, af can be a scalar or vector of length J.

Arguments describing effect size distribution:

pi: The probability that each variant has a direct effect on each trait. pi can be a scalar, vector of length K, or $J \times K$ matrix. If pi is a vector, each element corresponds to one trait. If pi is a matrix, each element corresponds to one variant-trait pair.

sporadic_pleiotropy: Logical argument controlling whether or not variants can have direct effects on multiple traits. If TRUE (default) causal variants are chosen for each trait independently with no restrictions. If FALSE, causal variants are chosen for each trait in order and are not allowed to directly affect multiple traits. This option must be set to TRUE if pi is a matrix. In some cases, using sporadic_pleiotropy = FALSE is inconsistent with other arguments, generating an error. For example if pi were set to 1, it would not be possible to avoid variants with causal effects on multiple traits.

pi_exact Logical argument controlling whether there is variation in the number of direct effect variants. This argument defaults to FALSE, in which case the number of causal variants will be random. If set to TRUE, the number of direct effect variants for trait k will be exactly equal to round(J * pi[k]). If pi is a matrix, this argument must be FALSE

snp_effect_function: An optional user-specified function or list of functions to generate direct effect sizes. SNP effect functions should take arguments n, sd, and snp_info and return a vector of n values with standard deviation sd. A function that does not return values with the specified variance (for example a deterministic function) can be used if h2_exact = TRUE. When this function is called by sim_mv, the snp_info argument will be given a data frame including the allele frequency and any variant annotations supplied to the snp_info argument of this function.

snp_info Optional dataframe of variant information to be passed to variant effect functions. If R_LD is specified, snp_info should have number of rows equal to the size of the supplied LD pattern. Otherwise snp_info should have J rows.

h2_exact : Logical argument controlling whether the realized heritability is exactly equal to the specified expected heritability.

Additional arguments:

est_s Logical argument. If TRUE, the function will sample estimates of standard errors. Defaults to FALSE.

2.2 Output Object

sim_mv produces an object of class sim_mv. Below, we describe each of the elements of a sim_mv object.
Simulated summary statistics

beta_hat : A $J \times K$ matrix of simulated effect estimates.

s_estimate : If est_s was set to true, this contains a $J \times K$ matrix of simulated standard errors

True variant effects

beta_marg : True marginal variant associations, equal to the expected value beta_hat.

beta_joint : True joint variant effects. If R_LD was missing then all variants are independent so beta_joint and beta_marg are equal.

se_beta_hat : True standard errors of beta_hat.

direct_SNP_effects_marg : True marginal direct variant effects.

direct_SNP_effects_marg : True joint direct variant effects. If there are no causal effects between traits or there is only one trait, then direct_SNP_effects_marg is equal to beta_marg and direct_SNP_effects_joint is equal to beta_joint.

Other useful information

direct_trait_effects : $K \times K$ direct trait effects matrix, equal to G provided to sim_mv (this is $D^{(dir)}$).

total_trait_effects : Total trait effects matrix, equal to $D^{(tot)}$.

trait_cor : Observational trait correlation.

R : Correlation of columns of beta_hat (equal to the identity if there was no sample overlap).

Sigma_G : $K \times K$ genetic variance-covariance matrix. Diagonal elements of Sigma_G give the realized trait heritability.

Sigma_E : $K \times K$ environmental variance-covariance matrix.

snp_info : Dataframe of variant information. At a minimum, snp_info contains allele frequencies of each variant. If snp_info was provided to sim_mv, this dataframe also contains any annotations included there.

3 Additional Simulation Accuracy Results

Figures 2,3,4, and 5 show quantile-quantile plots comparing the distribution of effect estimates and standard error estimates and products of these to distributions obtained by simulating summary statistics directly. These plots all support they hypothesis that summary statistics generated by GWASBrewer have the same distribution as summary statistics obtained by first simulating individual level genotype and phenotype data. R code implementing these simulations is included in Supplementary Code.

4 Computational Cost

4.1 Simulations for Time Cost Estimation

To estimate computational cost, we simulated data for one trait using GWASBrewer and then simulated individual level data using resample_inddata. We considered two LD patterns. In the first, AR1-500, a single block of 500 variants is repeated to cover the desired number of output variants. The single LD block is an AR1 matrix with correlation parameter 0.9. In the second pattern, EUR, we use the built-in LD pattern which contains 39 blocks with size ranging from 140 to 863 variants, covering a total of 19,490 variants. In all simulations, the proportion of causal variants is 0.01 and total heritability is equal to $10^{-7}J$, though these parameters should not affect the compute time meaningfully, except for the phenotype simulation step of individual-level data generation, which scales proportionally to the number of causal variants. We measured computational time using 'system.time()' in R, reporting the total CPU time consumed ('user'). For all parameter settings, we performed 10 replicates and report the median over all replicates. IQR is additionally provided in Supplementary Table S1.

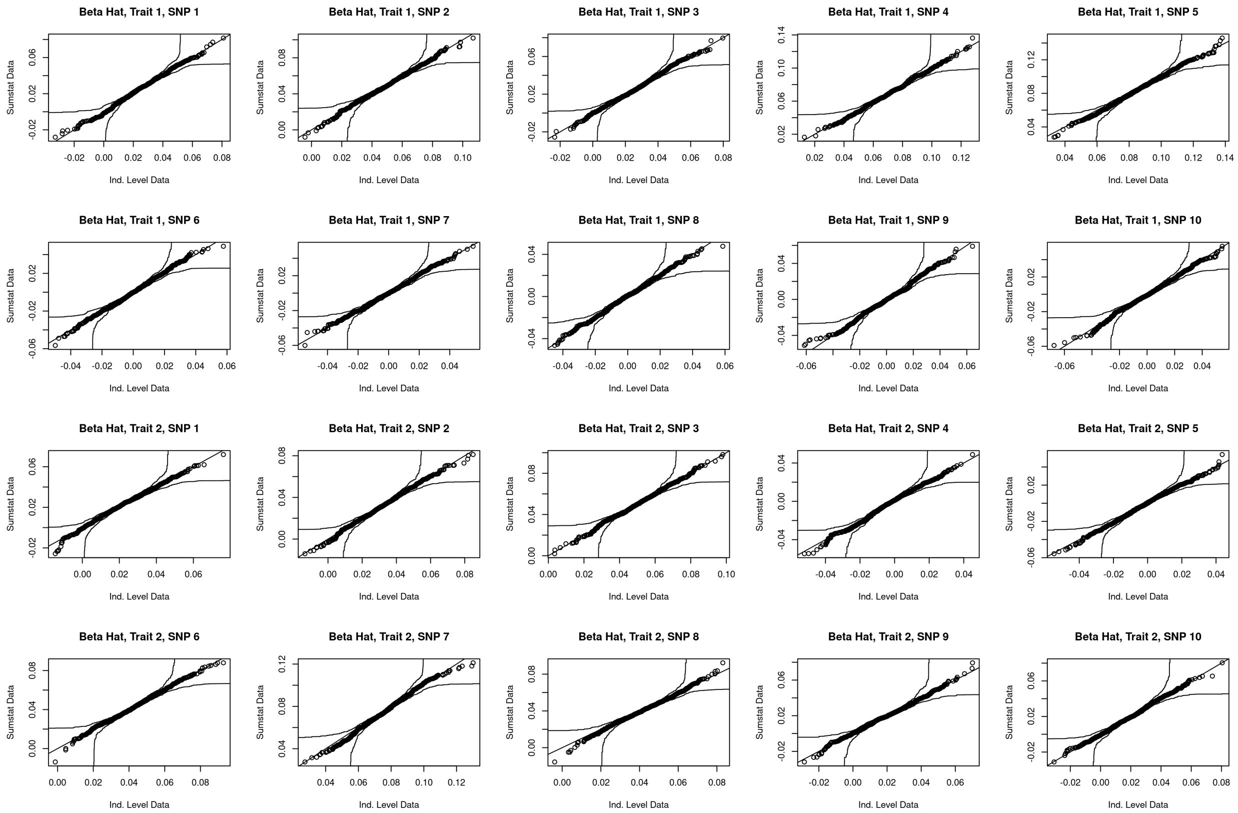

Figure 2: Q-Q plots comparing the distribution of effect estimates sampled directly and generated from individual-level data for each trait-variant combination.

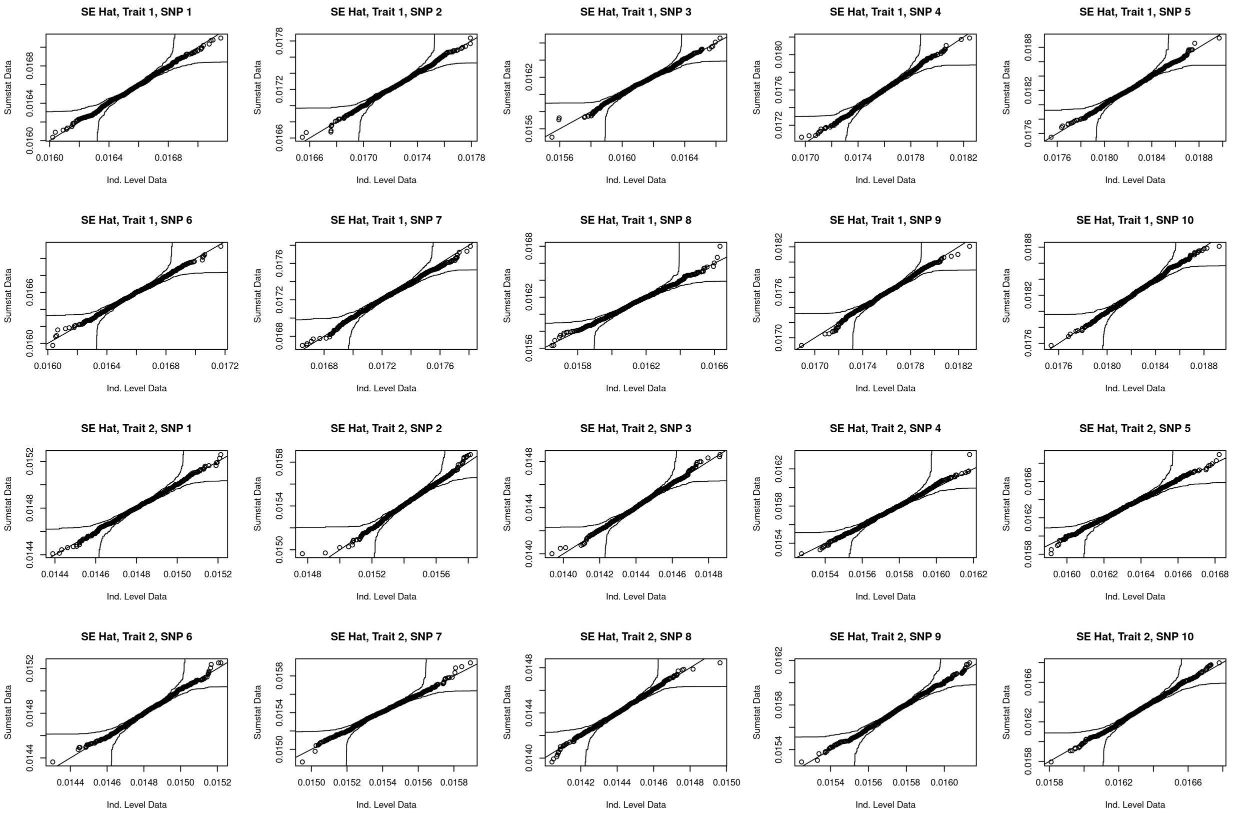

Figure 3: Q-Q plots comparing the distribution of standard estimates sampled directly and generated from individual-level data for each trait-variant combination.

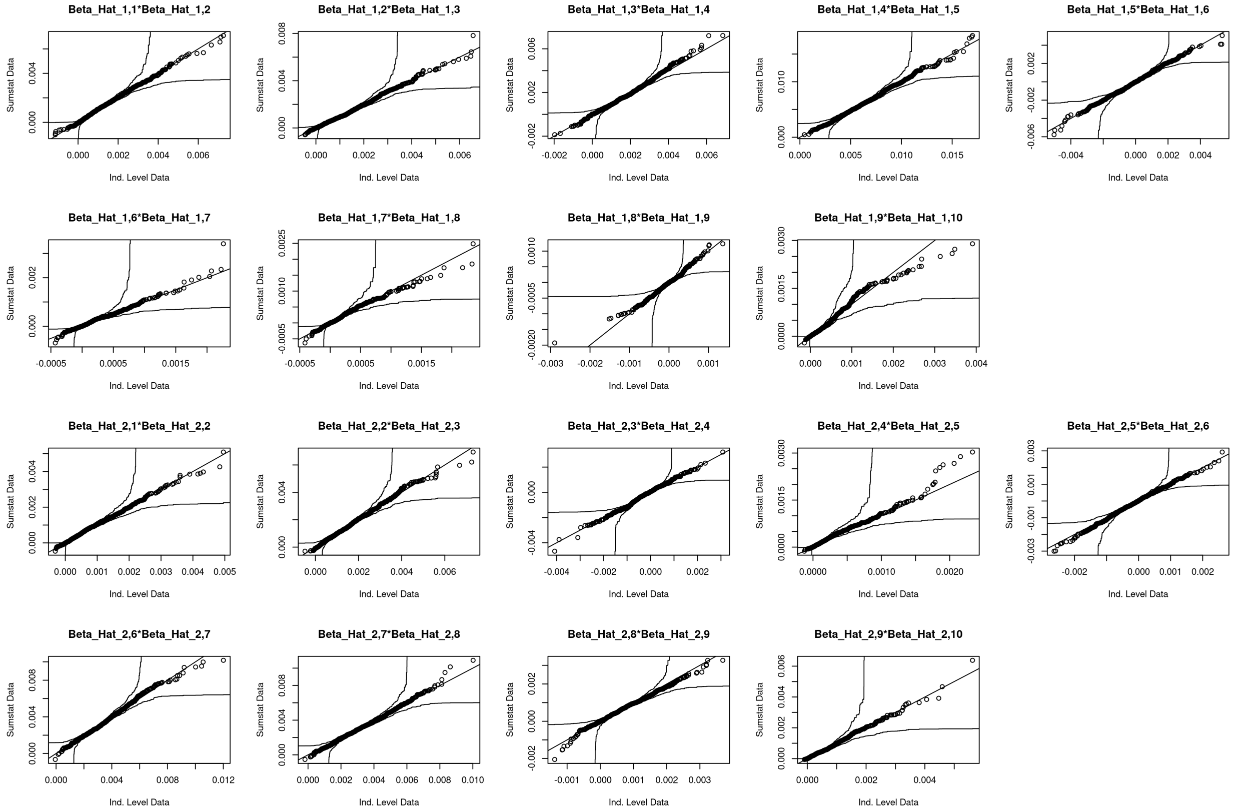

Figure 4: Q-Q plots comparing the distribution of products of effect estimates sampled directly and generated from individual-level data. Beta_Hat_i,j indicates the effect estimate for trait i and variant j.

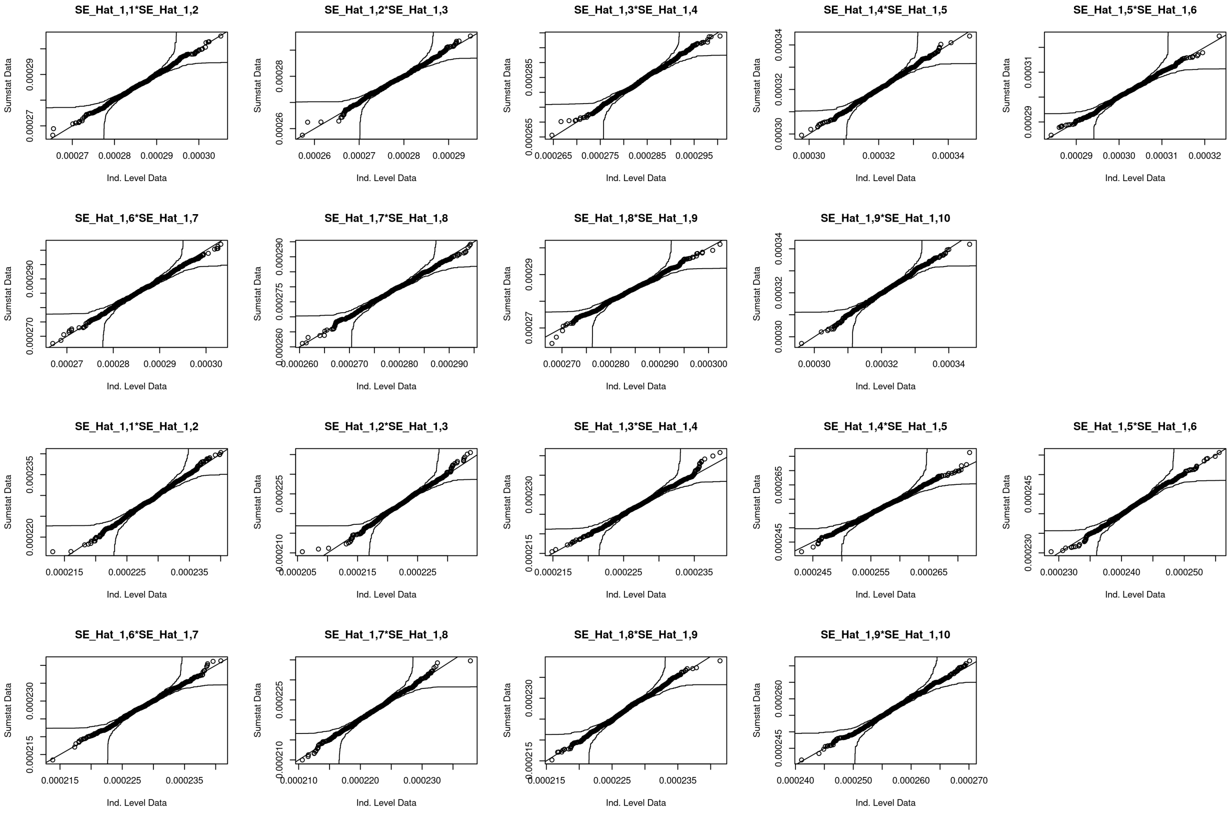

Figure 5: Q-Q plots comparing the distribution of products of standard error estimates sampled directly and generated from individual-level data. SE_Hat_i,j indicates the standard error estimate for trait i and variant j.

As shown in Main Text Table 1, we performed one set of simulations with 10,000 variants and sample size ranging between 100 and 5,000, and a second set with sample size equal to 100,000 and number of variants ranging between 500 and 5,000. In order to estimate the required time for individual-level data simulation with 100,000 samples and 1 million variants, we fit linear regressions to results from simulations with 100,000 samples, including number of variants as the only covariate. We then made predictions based on these models. The assumption that time scales linearly with number of variants is supported by our observed times and by our theoretical expectations.

All experiments were performed on a single personal computer running Ubuntu 22.04, with Intel i7-11700K @ 3.60GHz processor (8 cores, 2 threads per core), and 32G of RAM. Experiments were conducted in R 4.4.0 (compiled with gcc) through RStudio 2023.06.1 using BLAS/LAPACK 3.10.0 for matrix computations. Full outputs of `sessionInfo()` and `extSoftVersion()` are provided along with simulation code in Supplementary Code.
